# Identification of aurintricarboxylic acid as a selective inhibitor of the TWEAK-Fn14 signaling pathway in glioblastoma cells

**DOI:** 10.18632/oncotarget.14685

**Published:** 2017-01-17

**Authors:** Alison Roos, Harshil D. Dhruv, Ian T. Mathews, Landon J. Inge, Serdar Tuncali, Lauren K. Hartman, Donald Chow, Nghia Millard, Holly H. Yin, Jean Kloss, Joseph C. Loftus, Jeffrey A. Winkles, Michael E. Berens, Nhan L. Tran

**Affiliations:** ^1^ Department of Cancer Biology, Mayo Clinic Arizona, Scottsdale, Arizona 85259, USA; ^2^ Cancer and Cell Biology Division, The Translational Genomics Research Institute, Phoenix, Arizona 85004, USA; ^3^ Norton Thoracic Institute, St Joseph's Hospital and Medical Center, Phoenix, AZ 85004, USA; ^4^ Department of Biochemistry and Molecular Biology, Mayo Clinic Arizona, Scottsdale, Arizona 85259, USA; ^5^ Department of Surgery, University of Maryland School of Medicine, Baltimore, MD 21201, USA

**Keywords:** glioblastoma, survival, invasion, Fn14, aurintricarboxylic acid

## Abstract

The survival of patients diagnosed with glioblastoma (GBM), the most deadly form of brain cancer, is compromised by the proclivity for local invasion into the surrounding normal brain, which prevents complete surgical resection and contributes to therapeutic resistance. Tumor necrosis factor-like weak inducer of apoptosis (TWEAK), a member of the tumor necrosis factor (TNF) superfamily, can stimulate glioma cell invasion and survival via binding to fibroblast growth factor-inducible 14 (Fn14) and subsequent activation of the transcription factor NF-κB. To discover small molecule inhibitors that disrupt the TWEAK-Fn14 signaling axis, we utilized a cell-based drug-screening assay using HEK293 cells engineered to express both Fn14 and a NF-κB-driven firefly luciferase reporter protein. Focusing on the LOPAC1280 library of 1280 pharmacologically active compounds, we identified aurintricarboxylic acid (ATA) as an agent that suppressed TWEAK-Fn14-NF-κB dependent signaling, but not TNFα-TNFR-NF-κB driven signaling. We demonstrated that ATA repressed TWEAK-induced glioma cell chemotactic migration and invasion via inhibition of Rac1 activation but had no effect on cell viability or Fn14 expression. In addition, ATA treatment enhanced glioma cell sensitivity to both the chemotherapeutic agent temozolomide (TMZ) and radiation-induced cell death. In summary, this work reports a repurposed use of a small molecule inhibitor that targets the TWEAK-Fn14 signaling axis, which could potentially be developed as a new therapeutic agent for treatment of GBM patients.

## INTRODUCTION

Glioblastoma (GBM), or grade IV astrocytoma, is the most common primary malignant intracranial tumor in adults [1]. The current standard of therapy for newly diagnosed GBM is surgical resection with concurrent radiation and chemotherapy [2]. Despite aggressive treatment and recent clinical advances, the median survival from diagnosis is only 15 months and the survival statistics for patients have not improved significantly over the past three decades [3, 4]. This strongly highlights the need to establish novel therapeutic strategies.

Therapeutic failure and the consequent morbidity and mortality derive, in part, from the genetic heterogeneity and invasive proclivity of this tumor [5, 6]. Surgical resection eliminates the bulk tumor but the penumbra around the tumor border is a critical and challenging prognostic factor [7, 8]. Often following along myelinated white matter tracts, the neoplastic cells disperse from the tumor border and infiltrate the normal brain parenchyma [9–11]. This invasive sub-population is inherently resistant to cytotoxic therapy, which precludes effective clinical management of GBM and results in tumor recurrence [5, 12, 13]. Improved clinical treatment ultimately requires a thorough understanding of the signaling pathways that drive glioma invasion and the development of small molecule inhibitors specifically targeting the critical regulators.

Gene expression analysis of glioma cells migrating *in vitro* and invading *in vivo* has identified several gene candidates potentially involved in cell invasion and survival, including the tumor necrosis factor-like weak inducer of apoptosis (TWEAK) – fibroblast growth factor inducible 14 (Fn14) signaling axis [14, 15]. TWEAK is a multifunctional member of the tumor necrosis factor (TNF) superfamily of cytokines that is initially expressed as a transmembrane glycoprotein but can be proteolytically processed to its soluble form. TWEAK exerts its biological effects on cells via binding to the TNF receptor (TNFR) superfamily member Fn14, which is a type Ia transmembrane receptor lacking a cytoplasmic death domain. The TWEAK-Fn14 signaling axis plays an important role in regulating various aspects of tumor behavior such as growth, survival, invasion and angiogenesis [16–18]. Fn14 mRNA and protein expression is minimal to absent in normal brain tissue but increased with brain tumor grade and correlated with poor patient outcome [15, 19]. Activation of Fn14 enhanced glioma cell invasion and survival, which were mediated, in part, by Rac1 and NF-κB [19–24]. Thus, Fn14 plays a critical role in cancer cell invasion and survival and represents a potential therapeutic vulnerability in GBM. Currently, only one small molecule has been described in the literature that inhibits the TWEAK-Fn14 signaling cascade [25]. This molecule, L524-0366, prevents TWEAK: Fn14 engagement via binding to Fn14. However, L524-0366 is a tool compound and not suitable for clinical use. Thus, we developed a high throughput assay to screen for additional small-molecule inhibitors of TWEAK-Fn14 signaling and identified aurintricarboxylic acid (ATA) as a potent inhibitory compound. ATA inhibited TWEAK-induced Fn14 activation of downstream signaling pathways and suppressed glioma cell migration and invasion. Moreover, ATA suppressed TWEAK-induced glioma survival in the presence of genotoxic stress. Taken together, these data demonstrate that ATA may be a potential therapeutic agent to limit invasion and enhance chemotherapeutic drug efficacy in GBM.

## RESULTS

### High throughput screen identified aurintricarboxylic acid as a specific inhibitor of TWEAK-Fn14 signaling

**Figure 1:**
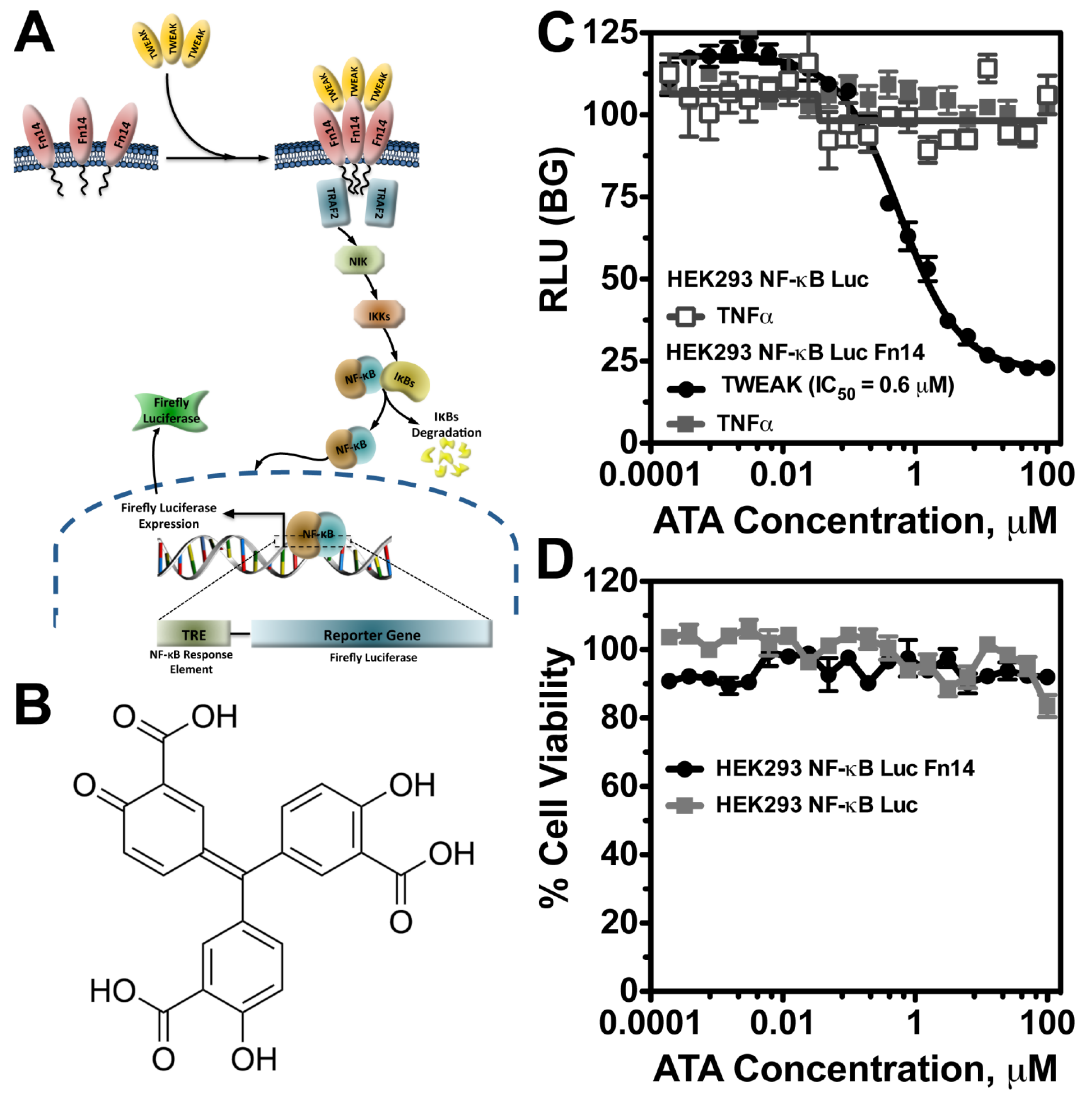
ATA inhibited TWEAK-Fn14-mediated NF-κB activation **A**. Schematic drawing of TWEAK-Fn14 signaling pathway leading to NF-κB-driven luciferase expression in reporter cell lines. **B**. Structure of ATA. **C**. Dose response curve of inhibitory activity of ATA in NF-κB-Luc and NF-κB-Luc/Fn14 cells following TWEAK or TNFα stimulation. **D**. ATA effects on NF-κB-Luc and NF-κB-Luc/Fn14 cell growth as measured by CellTiterGlo® assay.

Our *in vitro* and *in vivo* data establish the TWEAK-Fn14 signaling axis as an attractive target to enhance therapeutic efficacy in GBM [15, 19, 20]. TWEAK-Fn14 signaling has been implicated in the pathogenesis of multiple diseases, ranging from autoimmune disorders to cancer; however, to date, only one small-molecule inhibitor of TWEAK-Fn14 signaling has been reported [25]. To identify drug-like inhibitors of the TWEAK-Fn14 pathway, we developed a cell-based assay for high-throughput screening (HTS) using the LOPAC1280 library of 1280 pharmacologically active compounds. Since parental HEK293 cells express low levels of Fn14 and exhibit a minimal cellular response to exogenous TWEAK treatment [26, 27], we engineered HEK293 cells to overexpress Fn14 as well as a NF-κB-driven luciferase reporter. Stimulation with TWEAK is predicted to promote Fn14 trimerization, TNFR-associated factor (TRAF) recruitment to the Fn14 cytoplasmic tail, and downstream NF-κB activation [16]. Activated NF-κB then translocates to the nucleus and triggers firefly luciferase expression (Figure 1A). This cell-based assay interrogates allosteric modulators that can have a functional consequence throughout the TWEAK-Fn14 signaling pathway. In the preliminary drug-screening assay, we found that aurintricarboxylic acid (ATA) (Figure 1B) specifically inhibited TWEAK-Fn14-mediated NF-κB activation. Dose response curves of inhibitory activity of ATA in NF-κB-Luc and NF-κB-Luc/Fn14 cells following TWEAK or TNFα stimulation showed that ATA specifically inhibited only Fn14-driven NF-κB activation, with an IC_50_ of 0.6 μM (Figure 1C). ATA did not demonstrate any cytotoxic effects on NF-κB-Luc or NF-κB-Luc/Fn14 cells, which indicates the effect of ATA on TWEAK-Fn14 signaling is due to a specific pharmacological effect (Figure 1D).

### ATA suppressed TWEAK-Fn14-mediated NF-κB, Akt, and Src phosphorylation in GBM cells

**Figure 2:**
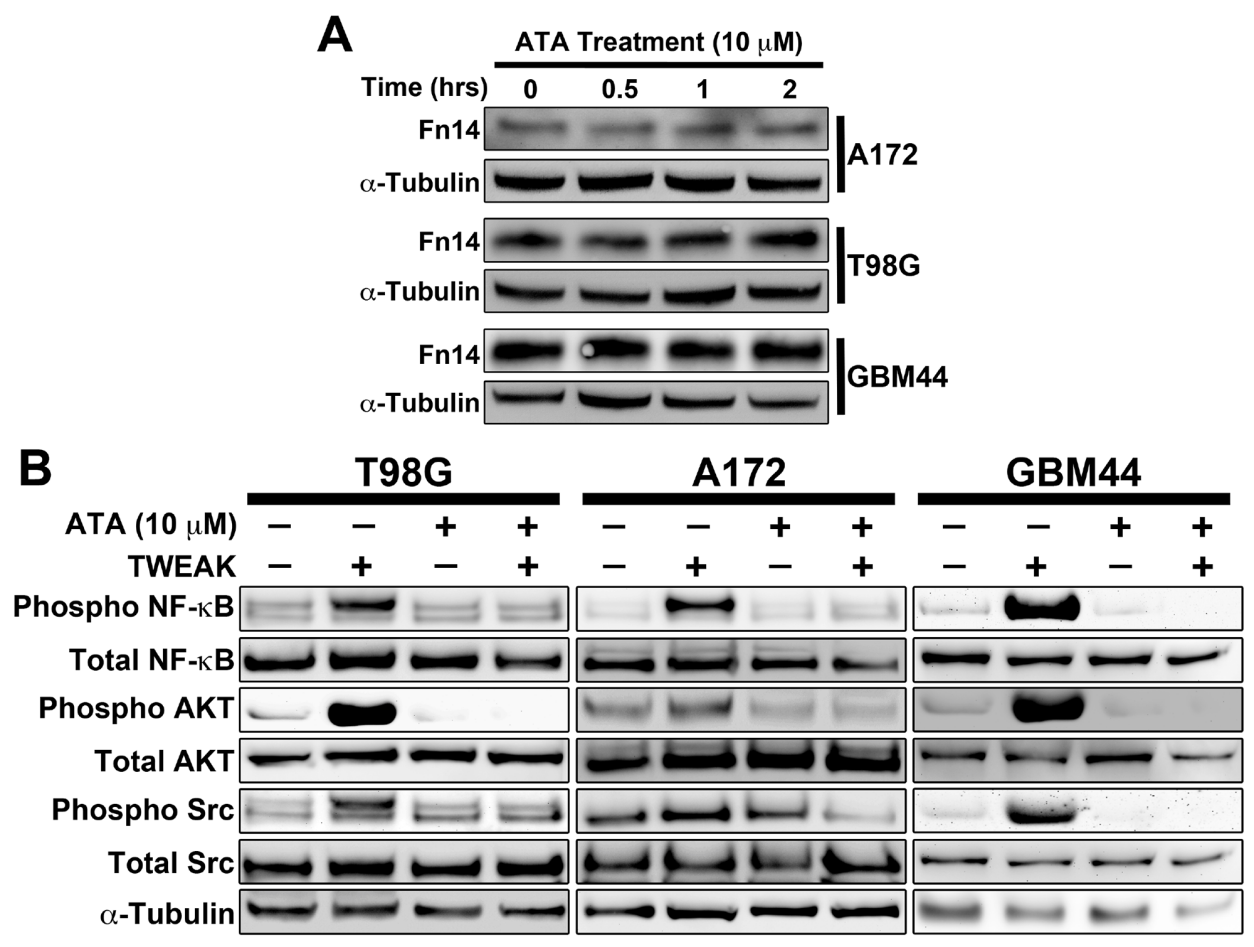
ATA suppressed TWEAK-Fn14 downstream pathway activation in glioma cells **A**. T98G, A172, and GBM44 glioma cells were treated with ATA (10 μM) for the indicated times, lysed, and Western blot analysis was performed using Fn14 and α-tubulin antibodies. **B**. T98G, A172, and GBM44 glioma cells were stimulated with TWEAK (100 ng/mL) in presence or absence of ATA (10 μM) for 10 min, lysed, and Western blot analysis was performed using Phospho NF-κB (p65), Total NF-κB (p65), Pan Phospho Src, Total Src, Phospho AKT, Total AKT, and α-tubulin antibodies.

Since ATA suppressed TWEAK stimulation of NF-κB activity in HEK293 cells, we next investigated the effects of ATA on signaling cascades downstream of Fn14 using two established glioma cell lines (T98G and A172) and the GBM patient-derived xenograft (PDX) line GBM44. Prior to initiating these studies, we first confirmed that ATA treatment of glioma cells did not alter Fn14 mRNA (data not shown) or protein (Figure 2A) levels. Treatment with ATA abrogated TWEAK activation of downstream signals including phosphorylation of the NF-κB family member p65, Akt, and Src in all three GBM cell lines (Figure 2B). Together, this data corroborates the role of ATA as an inhibitor of TWEAK-Fn14 signaling.

### TWEAK-stimulated Rac1 activity and TRAF2 recruitment to Fn14 is attenuated by ATA

**Figure 3:**
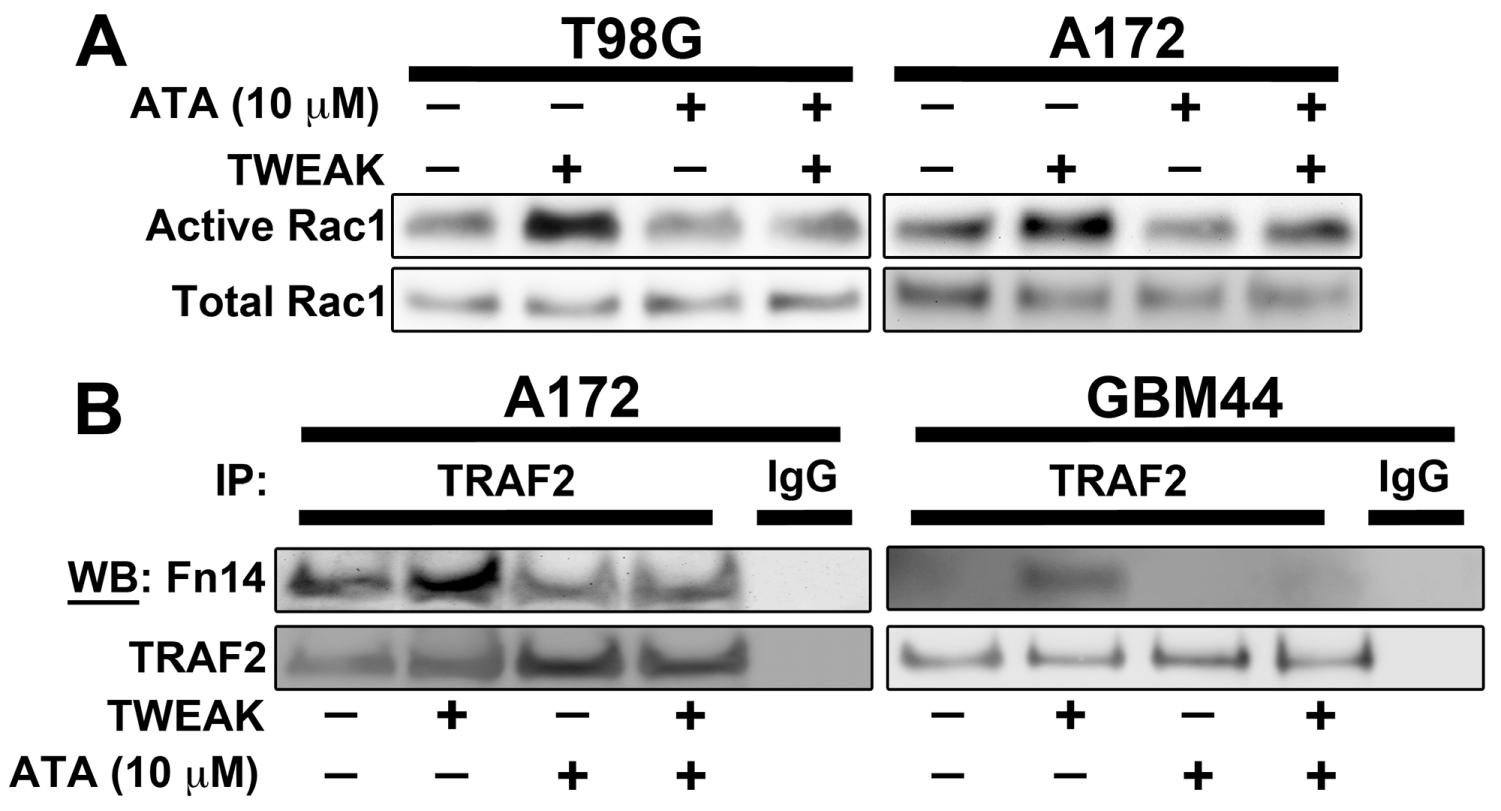
ATA blocked TWEAK-stimulated Rac1 activation and TRAF2 recruitment to Fn14 cytoplasmic domain in glioma cells **A**. T98G and A172 glioma cells were stimulated with TWEAK (100 ng/mL) in presence or absence of ATA (10 μM) for 10 min and lysed. Rac1 activity was determined using Rac1 activity assay kit. **B**. A172 and GBM44 glioma cells were stimulated with TWEAK (100 ng/mL) in presence or absence of ATA (10 μM) for 2 min and lysed. Cell lysates were immunoprecipitated using TRAF2 or IgG control antibody and then Western blot analysis was conducted using Fn14 and TRAF2 antibodies.

TWEAK-Fn14 signaling promotes the activation of Rac1 to regulate glioma cell migration and invasion [19, 22, 28]. Therefore, we utilized a Rac1 pulldown assay to examine the effect of ATA on TWEAK-induced Rac1 activation. ATA treatment effectively inhibited activation of Rac1 by TWEAK in the A172 and T98G cell lines (Figure 3A). TWEAK binding to Fn14 results in receptor trimerization, TRAF association with the Fn14 cytoplasmic tail, and the initiation of downstream signaling cascades that result in pleiotropic cellular responses [16]. We have demonstrated that a functional TRAF binding domain and recruitment of TRAF2 is required for TWEAK-dependent signaling, including activation of the NF-κB and SGEF pathways [20, 23, 29]. Since we observed that ATA decreases TWEAK activation of NF-κB, we next investigated if ATA alters TRAF2 recruitment to Fn14. A172 and GBM44 cells were stimulated by TWEAK in the presence or absence of ATA and TRAF2 binding to Fn14 was investigated via a co-immunoprecipitation assay. Immunoprecipitation of TRAF2 after TWEAK treatment showed an increased binding of TRAF2 to Fn14. However, ATA suppressed TRAF2 binding to Fn14 in both cell lines (Figure 3B). Co-precipitation of TRAF2 was verified in immunoprecipitations with an Fn14 antibody (data not shown).

### ATA inhibited TWEAK-Fn14 mediated GBM migration and invasion

**Figure 4:**
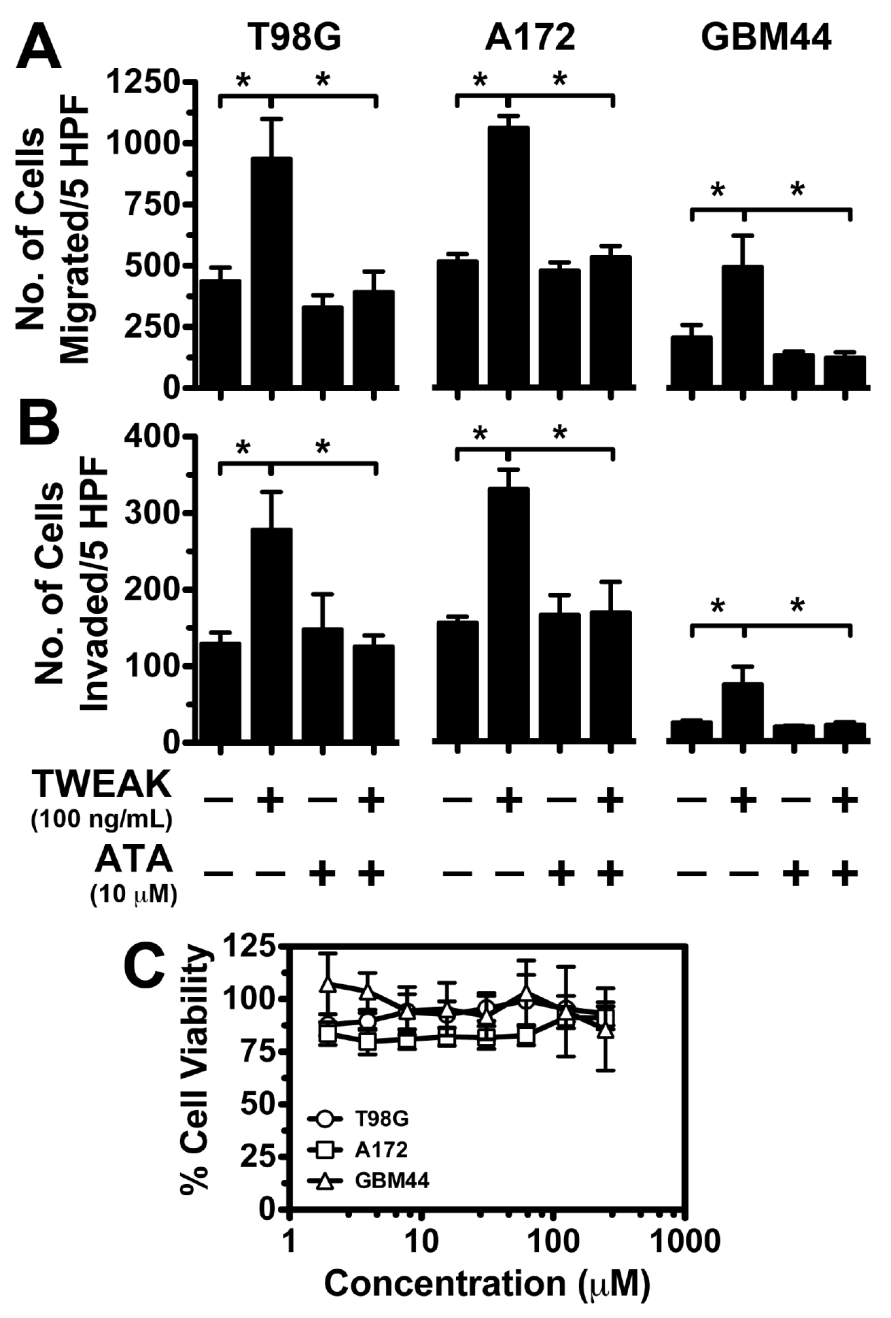
ATA repressed TWEAK-stimulated glioma cell migration and invasion without causing cell cytotoxicity **A & B**. A172, GBM44, and T98G glioma cells were added to the top well of a modified transwell chamber pre-coated with collagen in serum-free DMEM + 0.1% BSA to assess migration (A) or 10 mg/mL growth factor-free Matrigel to assess invasion (B). Either TWEAK alone (100 ng/mL) or TWEAK and ATA (10 μM) was added to the lower wells and the number of cells migrated to the bottom chamber quantitated after 5 hr. Values are mean ± standard deviation of triplicate measurements (*, p < 0.01). **C**. Glioma cells were seeded in 96-well plates and after 24 hr of incubation either vehicle (DMSO) or ATA at indicated concentration was added to each well. After 72 hr of incubation viability of the cells was measured using CellTiterGlo assay kit. Values are mean ± standard deviation of six separate measurements.

Previous studies have shown that knockdown of Fn14 or signaling axis proteins downstream of this receptor inhibit TWEAK-stimulated glioma cell chemotactic migration and invasion [15, 19, 22]. To corroborate these results with pharmacological inhibition of Fn14 signaling, we investigated if ATA suppressed TWEAK-dependent migration and invasion using transwell migration and Matrigel invasion assays, respectively. We observed that ATA significantly repressed TWEAK-induced glioma cell migration (Figure 4A) and invasion (Figure 4B) in T98G, A172, and GBM44 cell lines *in vitro* without altering cell viability (Figure 4C).

### ATA sensitized glioma cells to TMZ and radiation therapy

**Figure 5:**
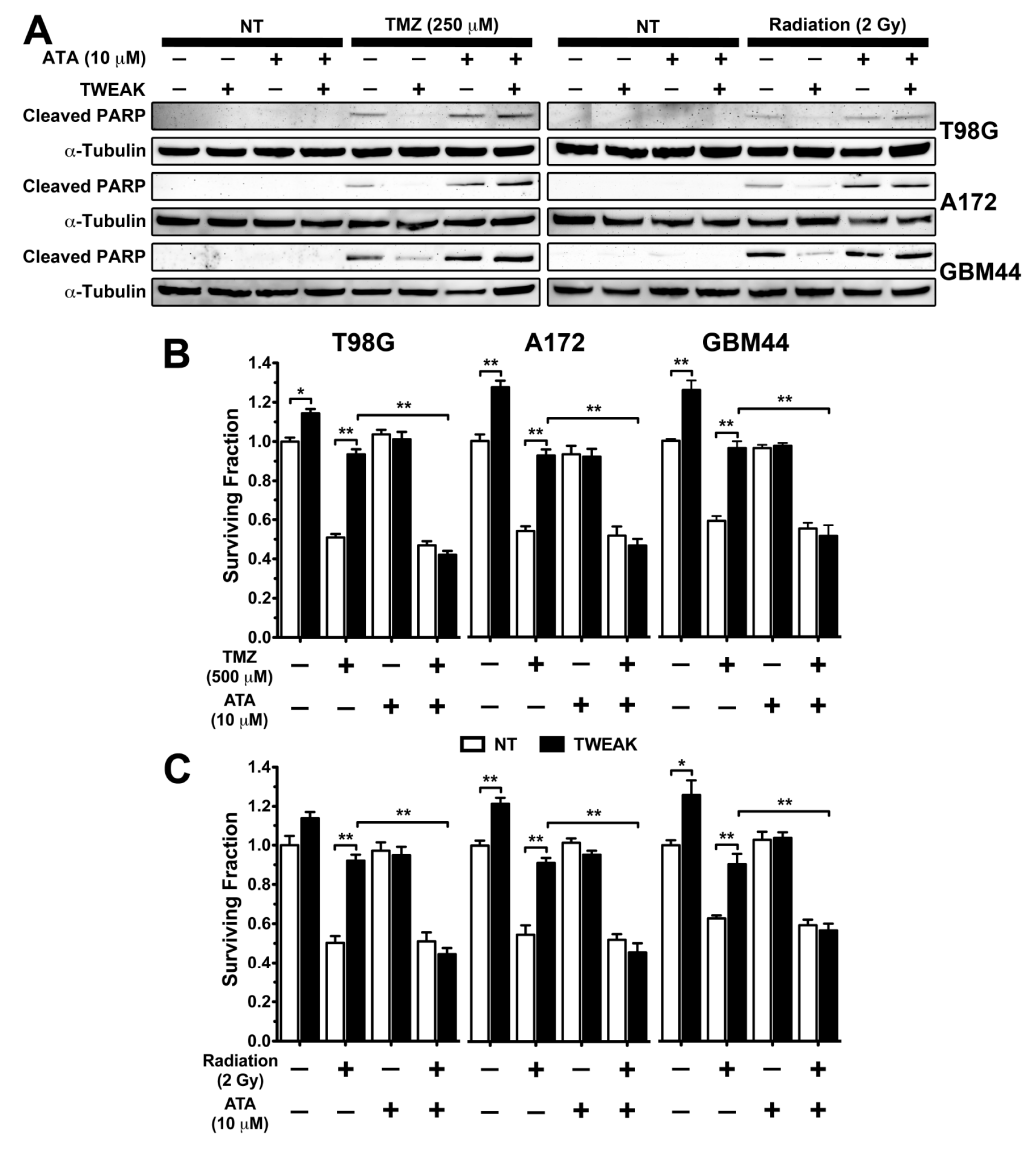
ATA suppressed TWEAK-stimulated glioma cell survival after TMZ and radiation therapy **A**. T98G, A172, and GBM44 glioma cells were treated with ATA (10 μM), TMZ (250 μM), ATA + TMZ, Radiation (2 Gy), and ATA + Radiation in presence or absence of TWEAK (100 ng/mL) for 48 hr and lysed. Western blot analysis was performed using cleaved PARP and α-tubulin antibodies. **B & C**. A172, GBM44, and T98G glioma cells were treated with ATA (10 μM), TMZ (500 μM), ATA + TMZ, Radiation (2Gy), and Radiation + ATA in presence or absence of TWEAK (100 ng/mL) for 24 hr. Cells were trypsinized and 250 cells were seeded in triplicate in 35 mm dishes and allowed to form colonies. At the end of the assay cells were fixed in PFA and stained with crystal violet, and number of colonies were counted. Values are mean ± standard deviation of three separate measurements (**p<0.01)

The invasive GBM cell subpopulation is radio-and chemo-resistant and we have shown that TWEAK stimulation of glioma cells *in vitro* suppresses apoptosis induced by cytotoxic therapy [5, 20]. To test if ATA can diminish the survival phenotype conferred by TWEAK, we treated T98G, A172, and GBM44 glioma cells with TWEAK and either TMZ or 2 Gy radiation in the presence or absence of ATA for 48 hours and used poly ADP ribose polymerase (PARP) cleavage as a marker of apoptosis. TWEAK treatment abrogated radiation- and TMZ-induced PARP cleavage. However, glioma cells treated with TWEAK and TMZ or 2Gy radiation with concurrent ATA showed elevated cleaved PARP levels compared to cells treated with TWEAK and TMZ or radiation (Figure 5A). To further investigate if ATA could rescue TWEAK-induced glioma survival in the presence of TMZ and radiation, we next treated cells with TWEAK and TMZ or radiation for 24 hours in the presence or absence of ATA and measured colony formation. Cells treated with ATA displayed significantly impaired colony formation after TWEAK and TMZ or radiation treatment as compared to cells treated with TWEAK and TMZ or radiation alone (Figure 5B and 5C). Taken together, these data validate that ATA inhibited the pro-survival phenotype conferred by TWEAK-Fn14 signaling following TMZ and radiation treatment.

### Fn14 depletion significantly improves glioma cell survival *in vivo*

**Figure 6:**
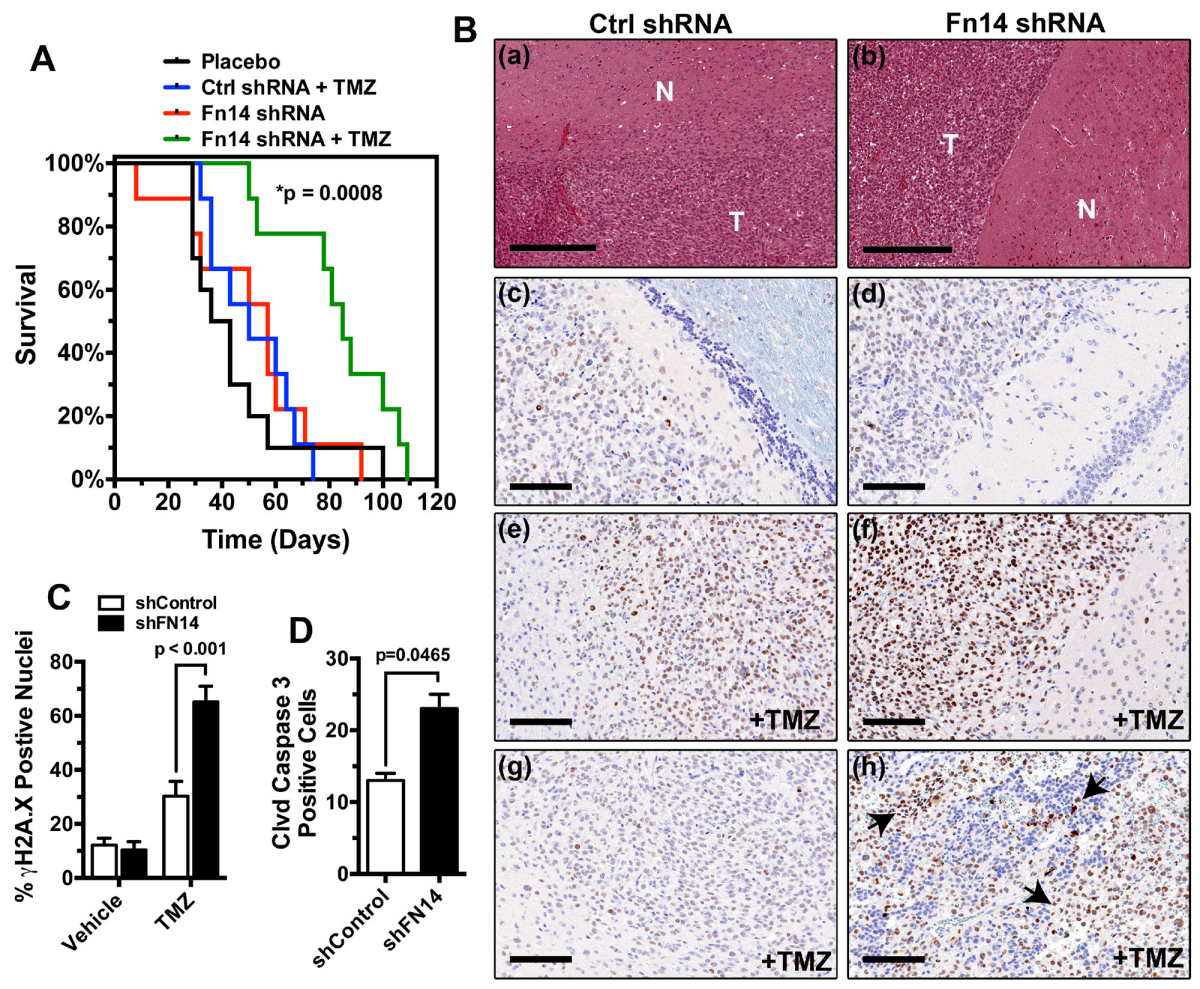
Knockdown of Fn14 expression concurrent with TMZ treatment significantly increases animal survival **A**. Kaplan-Meier survival curves of athymic nude mice with intracranial xenografts of GBM44 cells transduced with a control shRNA or shRNA targeting Fn14 and treated with/without TMZ(control shRNA + TMZ vs. Fn14 shRNA + TMZ p<0.0008). **B**. a, c, e, g = control shRNA; b, d, f, h = Fn14 shRNA. a, b) H&E staining of GBM44 PDX tumor (T) and normal (N) brain tissue. Bar=300 uM. c, d) pH2AX staining of vehicle-treated brain tissue.e, f) pH2AX staining of TMZ-treated brain tissue. g, h) Cleaved Caspase 3 staining of TMZ-treated tumor tissue. Arrows highlight apoptotic cells. Bars for c-h=100 uM. **C**. Quantification of γH2AX positive nuclei in tumors expressing a control shRNA or Fn14 shRNA and treated with the vehicle alone or TMZ. **D**. Quantification of cleaved caspase 3 positive nuclei in control shRNA or Fn14 shRNA tumors treated with TMZ.

The TWEAK-Fn14 signaling cascade induces glioma cell invasion and survival [15, 19–21, 30]. TWEAK stimulation results in the Fn14-dependent upregulation of BCL-xL and BCL-W proteins that confer chemoresistant properties to GBM cells [20]. Activated Fn14 also induces SGEF expression, which modulates the function of the DNA damage response protein BRCA1 to augment glioma cell survival in the presence of TMZ [21, 23]. Taken together, this data suggests that inhibition of the TWEAK-Fn14 signaling axis may limit glioma cell chemoresistance. To test if Fn14 depletion can act synergistically with TMZ *in vivo*, we utilized the patient-derived GBM44 cell line that is relatively resistant to TMZ therapy [31]. We generated stable GBM44 cell lines expressing a non-targeting shRNA or Fn14 shRNA, injected the cells intracranially into athymic nude mice, and monitored for survival in the presence or absence of TMZ treatment. We found that Fn14 depletion in conjunction with TMZ treatment significantly enhanced survival when compared to TMZ treatment alone (p < 0.0008; Figure 6A). Tumors with decreased Fn14 expression also showed significantly higher levels of markers of apoptosis, including γH2AX and cleaved-caspase 3 (Figure 6B-6D). This data demonstrates that inhibiting Fn14 expression, and thus function, can enhance chemotherapeutic vulnerability *in vivo*.

## DISCUSSION

The aggressive invasion of glioma cells into the surrounding brain parenchyma renders surgical resection incomplete and ultimately results in tumor recurrence. This invasive subpopulation is inherently resistant to chemotherapy and radiation and represents a challenge in the current clinical management of GBM. We have demonstrated a vital role for the TWEAK-Fn14 signaling axis in the regulation of GBM cell invasion and survival in the presence of cytotoxic therapeutics. Thus, exploiting or targeting TWEAK/Fn14 pathway components is an area of active research [16, 18, 32, 33]]. TWEAK binding to Fn14 activates the Src and Rac1 pathways that drive GBM invasion [19, 28]. Additionally, the TWEAK-Fn14 interaction results in the upregulation of anti-apoptotic Bcl family members and stimulates the AKT pathway to confer chemo-and radio-resistance to cells [20, 24]. These pro-tumorigenic phenotypes represent impediments to the efficacy of the current standard of care, and therefore, targeting this pathway may sensitize GBM cells to cytotoxic therapy.

The TWEAK and Fn14 ligand-receptor pair has been implicated in the maintenance and progression of multiple cancers, and efforts have focused on either activating or inhibiting this pathway [16, 18]. Preclinical and Phase I clinical trials have tested the efficacy of agonistic anti-Fn14 or antagonistic anti-TWEAK antibodies for multiple tumor subtypes, including breast carcinoma and melanoma [34–37]. In most cancer cell lines, overexpression and activation of Fn14 initiates pro-tumorigenic responses, not cell death [16, 18]. Thus, treatment with an agonistic Fn14 monoclonal antibody may potentiate Fn14-dependent tumorigenesis. Moreover, tumors with high expression of Fn14, including GBM and melanoma, often express low levels of TWEAK [15, 19, 32] (unpublished results). Fn14 overexpression may initiate TWEAK-independent signaling and this notion of TWEAK-independent Fn14 signaling presents a limitation to anti-TWEAK therapy [16, 18, 38]. Therefore, a small-molecule inhibitor that targets Fn14 or Fn14 downstream signaling nodes may be a more effective mode of therapy.

Here we identify the compound ATA as a novel inhibitor of TWEAK-Fn14 signaling. ATA selectively inhibited TWEAK-Fn14-NF-κB signaling but did not alter TNFα activation of NF-κB. Notably, ATA inhibited TRAF2 recruitment to Fn14 after TWEAK binding, a vital first step in the stimulation of downstream intracellular cascades. These data suggest that ATA interferes with an early step in the signaling process. Such possibilities include an interaction at the receptor-ligand interface that prevents TWEAK binding or an inhibition of ligand induced receptor oligomerization. As such, ATA inhibited TWEAK activation of the Src, AKT, NF-κB, and Rac1 pathways and suppresses downstream transcriptional expression of pro-survival genes including Bcl-XL, Bcl-w, and SGEF (data not shown). However, ATA treatment did not affect Fn14 or TRAF2 protein or mRNA expression, suggesting that regulation of these proteins is not the mechanism of action for ATA inhibition of the TWEAK-Fn14 signaling cascade. Future studies will be focusing on identifying the target(s) of ATA in modulating the TWEAK-Fn14 signaling cascade, and how this affects the DNA repair machinery after TMZ treatment. Importantly, ATA inhibited TWEAK-stimulated glioma cell chemotactic migration, invasion and chemoresistance. These data demonstrate that ATA is a potent inhibitor of the TWEAK-Fn14 signaling axis and can potentially be utilized to enhance the therapeutic response in GBM.

Studies into the mechanism of ATA have revealed a complex role in the regulation of signaling pathways that may differ based on the concentration of ATA used [39–43]. In lung carcinoma cells, ATA treatment inhibited HGF-induced c-Met phosphorylation and subsequent cell migration [44]. Interestingly, depletion of Fn14 abrogates MET-driven invasion in NSCLC and therefore inhibitors for both receptors have been a proposed combination mode of therapy [45]. An additional study identified ATA as an inhibitor of platelet derived growth factor (PDGF)-induced signaling pathways in neuroblastoma cells [39]. PDGF and its cognate receptors play a vital role in the biology of GBM [46]. Therefore, ATA may function as multi-receptor inhibitor in cancer.

The identification of ATA as a potential therapeutic agent for TWEAK/Fn14 –mediated GBM pathogenesis presents a viable option to limit clinical evasiveness of this lethal tumor. Fn14 is overexpressed in multiple solid tumors, and therefore, its clinical utility may span beyond GBM [16, 18, 33]. Future studies will focus on the efficacy of ATA as part of a therapeutic regimen in preclinical models of GBM, along with the use of ATA chemical structure as a foundation for further exploration of drug modification for improved GBM therapy.

## MATERIALS AND METHODS

### Screening of the LOPAC1280 Library

The LOPAC1280 library was purchased from Sigma-Aldrich (St. Louis, MO). To discover compounds that can inhibit TWEAK-induced Fn14 signaling, the LOPAC1280 library was screened against control and Fn14-overexpressing HEK293 reporter cell lines that express firefly luciferase upon NF-κB activation. These cell lines have been described previously [25, 26].

For screening, NF-κB-Luc and NF-κB-Luc/Fn14 reporter cells were seeded in 384-well plates at 3 × 10^3^ cells/well in 20 μL Opti-MEM media (Invitrogen) and incubated for 48 hr at 37˚C. After 48 hr incubation, 2.5 μL of each drug solution (250 μM and 500 μM stocks) in DMSO was added to the designated wells at a final concentration of 10 and 20 μM. After 1 hr of drug incubation at 37˚C, 2.5 μL of recombinant TWEAK (Peprotech Inc.; 300 ng/mL) in 0.1% BSA in PBS was added to each well and incubated for 8 hr at 37˚C. DMSO alone was used as a negative control whereas soluble recombinant Fn14-Fc protein (R & D System Inc., Minneapolis, MN) was used as a positive control for the assay. Luminescent signal was determined using Bright-Glo assay kit (Promega, Madison, WI) according to the manufacturer's instructions and normalized to negative control. Two separate counter-screen assays were carried out using TNFα to stimulate NF-κB activity in NF-κB-Luc and NF-κB-Luc/Fn14 reporter cells. The counter-screen assays were performed similar to the drug screening assay described above, except 2.5 μL of 10x purified recombinant TNFα (R & D Systems; 300 ng/mL) in 0.1% BSA in PBS was added to each well instead of TWEAK for NF-κB activation. Small molecule inhibitors that showed dose-dependent (10 μM and 20 μM) inhibition of the luciferase signal following TWEAK stimulation but not after TNFα stimulation were further validated by performing a dose response analysis. The selected small molecule inhibitors were tested at concentrations ranging from 0.0002 μM to 100 μM for their ability to suppress TWEAK-induced NF-κB activity in HEK293 NF-κB-Luc/Fn14 cells.

### Cell culture conditions

Human T98G and A172 glioma cell lines (American Type Culture Collection, Manassas, VA) were maintained in DMEM with high glucose (Invitrogen, Carlsbad, CA) supplemented with 10% heat-inactivated fetal bovine serum (FBS) (Invitrogen, Carlsbad, CA) in a 37°C, 5% CO_2_ atmosphere at constant humidity. The primary glioma patient derived xenograft (PDX) line GBM44 was derived from a patient surgical sample and maintained as a flank xenograft in immunodeficient mice [47, 48]. GBM44 flank tumor was resected and brought to an approximate single cell suspension via mechanical dissociation. The cells were then maintained in DMEM with high glucose supplemented with 10% FBS in a 37°C, 5% CO_2_ atmosphere at constant humidity. In the experiments with TWEAK stimulation, the cells were placed in serum reduced media (DMEM + 0.5% FBS) for 16 hr before stimulation with 100 ng/mL TWEAK (Peprotech, Rocky Hill, NJ).

### Western blot analysis

Cells were lysed in 2x SDS sample buffer (0.25 M Tris-HCl, pH 6.8, 10% SDS, 25% glycerol) containing 10 μg/mL aprotinin, 10 μg/mL leupeptin, 20 mM NaF, 2 mM sodium orthovanadate, and 1 mM phenylmethylsulfonyl fluoride. Protein concentrations were determined using the BCA assay (Pierce) with bovine serum albumin as a standard. Thirty micrograms of total protein was loaded per lane for SDS-PAGE. After 4°C transfer to nitrocellulose membranes (Invitrogen), membranes were blocked with either 5% nonfat milk or 5% BSA in Tris-buffered saline, pH 8.0, containing 0.1% Tween 20 (TBST) prior to addition of the following primary antibodies: Fn14, phospho-NF-κB p65 (S536), NF-κB p65, phospho-AKT (Y473), AKT, phospho-SRC (Y416), SRC, cleaved PARP, α-Tubulin, and β-Actin (Cell Signaling Technology, Danvers, MA) and followed with peroxidase-conjugated anti-mouse IgG or anti-rabbit IgG secondary antibody. Immunoreactive proteins were detected using SuperSignal West Dura Chemiluminescent Substrate (Thermo Scientific) with a UVP BioSpectrum 500 Imaging System (Upland, CA).

### Immunoprecipitation

A172 and GBM44 cells were pre-treated with 10 μM ATA (Sigma-Aldrich, St. Louis, MO) or vehicle for 1 hr before stimulation with 100 ng/mL TWEAK for 2 min. Subsequently, cells were lysed on ice in a buffer containing 10 mM Tris-HCl (pH 7.4), 0.5% Nonidet P-40, 150 mM NaCl, 1 mM phenylmethylsulfonyl fluoride, 1 mM EDTA, 2 mM sodium orthovanadate, 20 mM sodium fluoride, 10 μg/mL aprotinin, and 10 μg/mL leupeptin. Equivalent amounts of protein (1 mg) were pre-cleared and immunoprecipitated from the lysates using either TRAF2 antibody (Cell Signaling Technology, Danver, MA) or a control isotype-matched antibody, and then washed with lysis buffer followed by TX-100 buffer [10 mm HEPES (pH 7.4), 150 mm NaCl, 2 mm EDTA, 2 mm EGTA, 20 mm sodium fluoride, and 0.5% Triton X-100]. Samples were then re-suspended in 1x SDS sample buffer containing DTT and boiled, separated by SDS-PAGE, transferred to nitrocellulose for 1 hr at 4°C, and then proteins were detected using SuperSignal West Dura Chemiluminescent Substrate (Thermo Fisher Scientific).

### Clonogenic assays and apoptosis studies

Observations of colony forming capacity following cytotoxic insult were performed as described [49]. Briefly, T98G, A172 and GBM44 cells were treated with TMZ (500 μM) or radiation (2 Gy). In certain experiments, cells were additionally treated concurrently with TWEAK (100 ng/mL) or ATA (10 μM). Cells were trypsinized 24 hr post-TMZ treatment and plated in triplicate in 6-well cell culture dishes at 250 cells per well. Colonies were allowed to grow (approximately 6-7 days) before being fixed briefly in a 10% (v/v) methanol 10% (v/v) glacial acetic acid solution, stained with a 0.5% (w/v) crystal violet solution and washed with de-ionized water. Colonies were counted and surviving fractions were determined relative to the non-treated control for each cell line.

For apoptotic studies, T98G, A172, and GBM44 cells were treated with TMZ (250 μM), TWEAK (100 ng/mL), and ATA (10 uM) for 48 hr and whole cell lysates were analyzed for cleaved PARP by Western blot analysis.

### Cell viability assays

The CellTiterGlo® (Promega, San Luis Obispo, CA) assay was used to assess the cell viability after ATA treatment as previously described with minor modifications [50]. Briefly, T98G, A172, and GBM44 cells were seeded at a density of 3000 cells/well (80 μL) in 96 well plates. Increasing concentrations of ATA were added to the different wells (8 replicate wells for each condition) and incubated for 72 hr in 37°C, 5% CO_2_ atmosphere. Subsequently, CellTiterGlo® reagent was added to each well and luminescence was measured using Perkin Elmer Envision 2104 Multilabel Reader. On all 96 well plates, wells containing vehicle only or the positive control compound MG132 (a proteasome inhibitor toxic to most cell lines at 2 μM) were also included. Raw values were normalized on a plate-by-plate basis such that 100% cell viability was equivalent to the mean of vehicle wells and 0% cell viability was equivalent to the mean of the MG132 positive control. The normalized data was used to assess viability of glioma cells after ATA treatment.

### Chemotactic migration and matrigel invasion assays

Glioma cells (5.0 × 10^5^) were seeded in 100-mm diameter culture dishes and incubated overnight at 37°C. Subsequently, cells were incubated in DMEM, 0.5% FBS, 0.1% BSA for additional 16 hr at 37°C. For chemotactic migration assays, cells were harvested and added in triplicate to collagen-coated transwell chambers and allowed to migrate towards TWEAK (100 ng/mL) and/or TWEAK (100 ng/mL) and ATA (10 μM). For invasion assays, cells were harvested, resuspended in growth factor reduced Matrigel (Becton Dickinson, San Jose, CA) (1.0 × 10^5^ cells/50uL), added in triplicate to collagen-coated transwell chambers, and allowed to invade through Matrigel towards TWEAK (100 ng/mL) and/or TWEAK (100 ng/mL) and ATA (10 μM). After incubation for 24 hr at 37°C, non-invaded cells were scrapped off the upper side of the membrane and cells invaded to the other side of the membrane were fixed with 4% paraformaldehyde (PFA) and stained with ProLong® Gold Antifade reagent with DAPI (Invitrogen, Carlsbad, CA). Nuclei of invaded cells were counted in five high power fields (HPF) with a 20X objective.

### Rac1 activation assay

Rac1 activity assays were performed according to the manufacturer's protocol (Thermo Scientific, Rockford, IL). Briefly, A172 and GBM44 glioma cells (5.0 × 10^5^) were seeded in 100-mm diameter culture dishes and incubated overnight at 37°C. Subsequently, cells were incubated in DMEM. 0.5% FBS, 0.1% BSA for additional 16 hr at 37°C. Cells were then pre-incubated with 10 μM ATA or vehicle for 1 hr prior to 10% FBS stimulation for 2-10 min. Cell lysates were harvested and equal concentrations of protein were assessed for Rac1 activation.

### *In vivo* studies

Animal studies were approved by the Translational Drug Development Management Animal Care and Use Committee (Scottsdale, AZ). Glioma xenografts were established in athymic mice (Taconic) as described previously [51]. Briefly, 3 × 10^5^ glioma cells (GBM44 glioma cells expressing control shRNA or Fn14 shRNA) were intracranially implanted in the right striatum of athymic mice. Mice with established tumors were randomized into two treatment groups: vehicle control (n = 10) and TMZ (50 mg/kg) (n = 10). Treatment was given for 5 days through oral gavage. Mice were observed daily and euthanized upon reaching a moribund state and the brains were removed, rinsed, fixed in 10% neutral buffered formalin for 48 hours, and paraffin embedded using routine procedures. The survival was evaluated using Kaplan Meier survival plots and the log-rank test was used to determine statistical significance.

### Immunohistochemistry

FFPE samples were sectioned using standard procedures and adhered to charged microscope slides. Five μM sections underwent heat induced epitope retrieval and H&E and immunohistochemical (IHC) staining of tissue was performed using previously published procedures [52]. Antibodies to γH2AX and cleaved caspase 3 were purchased from Cell Signaling. Slides were scanned using the Aperio system (Leica Biosystems, Buffalo Grove, IL) and images collected at a magnification of 20×.

### Statistical analysis

Statistical analyses were done using the two-sample *t* test. P < 0.05 was considered significant.

## Abbreviations

GBM: glioblastoma
TWEAK: tumor necrosis factor-like weak inducer of apoptosis
Fn14: fibroblast growth factor-inducible 14
TNFRSF: tumor necrosis factor receptor superfamily
TMZ: temozolomide
ATA: aurintricarboxylic acid
